# The Roles of Exercise and Yoga in Ameliorating Depression as a Risk Factor for Cognitive Decline

**DOI:** 10.1155/2016/4612953

**Published:** 2016-12-01

**Authors:** Danielle C. Mathersul, Simon Rosenbaum

**Affiliations:** ^1^War Related Illness and Injury Study Center, Veterans Affairs Palo Alto Health Care System, Palo Alto, CA 94304, USA; ^2^School of Medicine, Stanford University, Stanford, CA 94305, USA; ^3^Department of Psychology, University of Pennsylvania, Philadelphia, PA 19104, USA; ^4^School of Psychiatry, Faculty of Medicine, University of New South Wales, Sydney, NSW 2052, Australia; ^5^The Black Dog Institute, Prince of Wales Hospital, University of New South Wales, Randwick, NSW, Australia

## Abstract

Currently, there are no effective pharmaceutical treatments to reduce cognitive decline or prevent dementia. At the same time, the global population is aging, and rates of dementia and mild cognitive impairment (MCI) are on the rise. As such, there is an increasing interest in complementary and alternative interventions to treat or reduce the risk of cognitive decline. Depression is one potentially modifiable risk factor for cognitive decline and dementia. Notably, exercise and yoga are two interventions known to both reduce symptoms of depression and improve cognitive function. The current review discusses the efficacy of exercise and yoga to ameliorate depression and thereby reduce the risk of cognitive decline and potentially prevent dementia. Potential mechanisms of change, treatment implications, and future directions are discussed.

## 1. Introduction

Globally, the population is aging, and disorders associated with cognitive decline, including dementia and mild cognitive impairment (MCI), are on the rise. Research is increasingly aiming to identify modifiable risk factors for cognitive impairment. Psychological disorders such as mood and anxiety disorders are highly prevalent in older populations [1]. These disorders are associated with cognitive deficits, including poor concentration, attention, and memory [2–4]. At the same time, there is growing evidence to suggest that mood and anxiety disorders may be risk factors for cognitive decline in later life [5]. Indeed, in older adults, dementia is associated with similar, overlapping symptoms to depression and delirium, and careful differential diagnosis is required to accurately confirm that [6, 7]. In this way, amelioration of low mood and depression may assist in improving symptoms of poor cognition in individuals either with or without a diagnosis of dementia or MCI. Beyond this though, these findings suggest that treatment and prevention of mood disorders prior to the onset of older age and/or cognitive decline may prevent or at least slow the development of clinically significant deficits in cognitive function.

Recent systematic reviews and meta-analyses have confirmed that both mid- and late-life depression are associated with an increased risk of dementia [8–10]. Indeed, the risk of Alzheimer's disease (AD) is almost doubled, and the risk of vascular dementia (VaD) is almost tripled, if an individual presents with clinical symptoms of depression in mid- to late-life [8–10]. Due to the high rates of cooccurrence between depression and dementia there is also the suggestion that depression may be part of the dementia prodrome. Interestingly, emerging evidence suggests that first presentation of depression later in life may reflect prodromal AD, whereas chronic or recurrent life-time depression may be associated with long-term cerebrovascular changes that predispose an individual towards the development of dementia, including VaD [8, 11] and AD [10, 12]. Furthermore, the presence of depression in individuals showing signs of cognitive decline (i.e., MCI) increases the risk of developing AD [13].

Depression is therefore a potentially modifiable risk factor for cognitive decline and dementia [14, 15]. Current first-line treatments (pharmaceutical and psychological interventions) demonstrate moderate success; however, nonresponse occurs in up to 50% of cases and many individuals fail to maintain long-term improvements [16–19]. Similarly, there is no cure for dementia and no treatment to reverse cognitive decline [20]. At best, some treatments help to alleviate certain cognitive or behavioral symptoms [21]. This highlights the need to investigate complementary and alternative treatments. Alternative therapies are often appealing to individuals who have found little or no improvement through standard interventions. Advantages of these interventions include reduced cost [22] (allowing greater access to at-risk populations and potentially producing a more sustainable long-term intervention), fewer side-effects, high levels of acceptability, and high congruence with popular culture (potentially increasing compliance). Beyond this, complementary and alternative interventions may have the potential to prevent future symptoms, in a way that is not evident with pharmaceutical interventions. In this review, we discuss the potential for two complementary and alternative interventions, exercise and yoga, to target depression and therefore reduce a significant risk factor for cognitive decline and dementia.

## 2. Exercise to Treat Depression

Both physical activity and exercise (i.e., a subset of physical activity that is planned, structured, and repetitive and has physical fitness as a final or an intermediate objective [23]) have been identified as having prevention and treatment effects on depression [24–26]. Moderate intensity, supervised exercise programs of at least nine-week duration (trials typically range from 8 to 16 weeks), utilising a combination of aerobic and resistance based exercise sessions, and a group format appear to have the greatest impact on reducing depressive symptoms [27, 28]. The largest community-based trial of exercise for major depressive disorder (MDD) involved 946 outpatients randomized to either 12 weeks of prescribed exercise, Internet-based cognitive behavioral therapy (CBT), or usual care consisting of brief CBT-focused therapy and antidepressant treatment and found that supervised exercise three times per week resulted in significantly lower depression severity compared to usual care and was equivalent to the Internet-CBT group [29]. These results are promising from an implementation perspective, especially for those groups for whom significant barriers to accessing standard clinician-delivered CBT exist, including stigma and financial costs associated with seeking treatment.

Nonetheless, there is ongoing debate within the scientific literature regarding the magnitude of effect of exercise on depression. Methodological differences between recent systematic reviews and meta-analyses [30], specifically around (i) the argument for and against the inclusion of pragmatic interventions such as physical activity counseling and yoga, which many argue better reflect real world clinical practice [31], and (ii) a large control group response (SMD −0.9) across exercise and depression RCTs [32], have resulted in an underreporting of the overall effect size. A 2016 meta-analysis of 25 RCTs including people with a diagnosis of depression and those with depressive symptoms found a large and significant overall effect on depression after adjustment for publication bias with an SMD of −1.11, corresponding to an approximate 5-point reduction in the Hamilton Rating Scale for Depression (HAM-D) and a greater than 6-point reduction in the Beck Depression Inventory (BDI), both of which are considered clinically significant based on the National Institute for Health and Care Excellence (NICE) guidelines [24, 33]. Additionally, it was found that a total of 1,057 negative studies would be required to nullify the significance of the main analysis, further justifying the robustness of the analysis [24]. These data are consistent with a 2015 meta-analysis of 23 RCTs investigating the effect of exercise on depressive symptoms of adults with neurologic disorders including AD which found a small but significant overall effect size of 0.28, with stronger effects of interventions meeting recommended physical activity guidelines [34]. In a subsequent 2016 meta-analysis, Schuch et al. reviewed the literature relating to exercise for depression in older adults (60 years) and found a large, significant effect of exercise on depression across the eight included RCTs (SMD = −0.90) [27].

In addition to large treatment effects, the preventative role of physical activity is becoming increasingly clear. A 2013 review of 25 studies found that baseline physical activity was negatively associated with a risk of future depression, with evidence for an effect with even low levels of physical activity (i.e., less than the recommended weekly total of 150 minutes [35]) [25]. Evidence for the bidirectional relationship between physical activity and depression continues to increase, with a recent longitudinal study of more than 1,000 participants showing that positive lifestyle behaviors (e.g., physical activity) at baseline were associated with a 22% (RR 0.76) reduced risk of episodes of a mood disorder at five-year follow-up [36]. These data are in line with a 2014 meta-analysis of observational studies finding that sedentary behavior, independent of physical activity, is associated with a significantly increased risk of depression [37]. This then becomes a vicious cycle, as people experiencing depression engage in significantly less physical activity compared to the general population [38, 39].

## 3. Exercise to Improve Cognitive Function

Exercise is increasingly acknowledged as having a positive effect on cognition in both the general and clinical populations, including those with depression. Evidence suggests exercise has modest effects on aspects of cognitive performance, including attention, processing speed, memory, and executive functioning [40]. While there is emerging evidence of a dose-response relationship between exercise and cognition [41], even low doses of exercise appear to provide attention and visuospatial benefits for both healthy and depressed older adults [42, 43]. For example, improvements in attention and inhibitory control but not working memory have been found following a single, moderate intensity (65–75% of estimated maximal heart rate) exercise session [43]. Subsequent analysis of data from a large study of exercise for depression found that individuals with MDD who received a public health guideline concordant dosage of exercise (equivalent to 30 min of moderate intensity aerobic exercise, five or more days per week) had improved psychomotor speed, attention, visual memory, and spatial planning at 12-week follow-up [41].

In addition to aerobic exercise there is promising and increasing evidence for the role of strength (resistance) training in promoting cognition among older adults. In arguably one of the most comprehensive studies to date, Suo et al. found that, among 100 older individuals (mean age 70.1 years) with MCI, six months of progressive resistance training but not computerised cognitive training significantly improved global cognition [44]. Interestingly, the computerised cognitive training program but not the exercise intervention attenuated decline in overall memory performance. The available evidence suggests that targeted exercise programs are feasible, acceptable, and effective in improving aspects of cognition among vulnerable populations including older adults.

## 4. Yoga to Treat Depression

Yoga is an integrative mind-body practice that combines physical activity (postures or asanas) with mindfulness practices (breath control (pranayama) and meditation (dhyana)), typically performed concurrently. Several recent systematic reviews and meta-analyses have demonstrated that yoga is effective in ameliorating symptoms of depression across a range of different clinical disorders, including MDD [45–48], posttraumatic stress disorder (PTSD) [49], stress and anxiety [50], and schizophrenia [51], as well as at-risk samples, such as pregnant women [52, 53], and individuals with chronic illnesses (multiple sclerosis [54], cancer [55], and fibromyalgia [56, 57]). Yoga also improves sleep [58], a known transdiagnostic and risk factor for psychopathology [59, 60]. Importantly, one study demonstrated significant improvements in depressed mood, well-being, and self-efficacy for a group of older adults (65–92 years), compared to either a control or exercise group [61]. Similarly, yoga improved mental/emotional wellness in a small group of older adults (66 years) [62]. These studies suggest that yoga may have beneficial effects at any age, though longitudinal studies are needed to determine whether there is a crucial period for prevention of cognitive decline.

A major confound within the literature is the heterogeneity in methodology, including type of yoga intervention (particularly in the ratio of asana versus pranayama), control group, and duration and intensity of the intervention. In particular, yoga treatment studies do not always offer dosage equivalency to other standard treatments for depression [45]. While yoga appears to demonstrate a significant advantage in comparison to a no treatment or passive control group, findings are less conclusive when compared to active controls. For example, one study found yoga to be less effective than electroconvulsive therapy (ECT) but at least equivalent to antidepressants [63]. In contrast, a recent systematic review and meta-analysis found in favour of yoga when compared to aerobic exercise or usual care (including group therapy, support group, or pharmaceutical intervention, but also wait-list control) [47]. While some studies suggest a cumulative psychological advantage in long-term meditators [64, 65] and lower rate of depression remission at 9-month follow-up for group yoga [66], overall, there is a paucity of longitudinal follow-up studies investigating the long-term effects of yoga.

## 5. Yoga to Improve Cognitive Function

A recent meta-analysis concluded that yoga is associated with overall moderate improvements in cognitive function, particularly attention, processing speed, executive function, and memory [67]. Acute intervention studies demonstrated more consistent cognitive improvements and stronger effects sizes than RCTs (*g* = .56 versus *g* = .33, resp.). Similarly, a recent systematic review confirmed that yoga improves executive function in both healthy individuals and those with chronic illnesses such as multiple sclerosis or type 2 diabetes mellitus [68]. A cross-sectional study of older adults (>55 years) classified as long-term yoga practitioners (>10 years) demonstrated significantly superior performance on multiple tests of attention than an age- and education-matched comparison group [69]. Importantly, yoga significantly improved cognitive impairments* and* depressed mood in a group of individuals with MDD [70], demonstrating scope for treatment across multiple domains. However, as with the literature regarding yoga interventions for mood difficulties, major limitations exist in the literature for yoga as a cognitive enhancing intervention. Very few studies provide sufficient information regarding the yoga intervention and the proportion of time spent practicing each of the major elements (i.e., asanas, pranayama, and dhyana) [67]. At the same time, yoga is inherently a mind-body exercise, and arguably, it is difficult to completely disentangle the various components (i.e., part of performing an asana correctly is to also simultaneously and concurrently employ pranayama and dhyana). Nonetheless, there is a need to compare yoga to interventions that are not inherently mind-body, that is, physical activity/exercise interventions alone, and to mindfulness-based interventions alone. A 2010 review of 10 studies comparing yoga to physical exercise found that yoga and exercise were equally beneficial for physical health, but yoga was superior to exercise for improving cognitive function and mental health [71]. However, further research is needed, particularly on populations with clinical disorders (either mental or cognitive) and in older samples. Currently, there is little regulation or standardisation in terms of recommended dose (e.g., session duration, frequency/regularity, and long-term duration) or type (especially in terms of variations in emphasis on the major components) of yoga intervention.

## 6. Mechanisms of Action

Many theorists suggest that the association between depression and dementia is due to a common underlying vulnerability factor (stress), which triggers neuroinflammation and disruption of the hypothalamic-pituitary adrenal (HPA) axis [72–75]. Resulting hypercortisolism may lead to neural damage, including reduced hippocampal neurogenesis [76, 77], possibly via reductions in hippocampal brain-derived neurotrophic factor (BDNF) [78, 79]. Hypercortisolism is associated with depression [80], long-term impairments in cognitive function [81], and dementia [82]. Abnormal expression of BDNF and associated reductions in hippocampal neurogenesis are linked to depression [78, 83]. Similarly, dysfunction of hippocampal neurogenesis [84] and reduced BDNF [85] are both associated with increased risk for dementia. More recently, a 38-year follow-up study found that mid-life neuroticism is associated with an increased risk of AD [86]. This is particularly interesting given that current theories propose a common, nonspecific factor of negative affect (neuroticism) as a vulnerability factor for emotional disorders, including depression and anxiety [87, 88]. That is, either stress or neuroticism may be vulnerability factor for depression and/or dementia. However, there is also evidence for a direct link between the two; depression may trigger neuroinflammation that then sensitises the brain to other precipitants of dementia [73]. Indeed, chronic neuroinflammation creates a vicious cycle that may maintain depression and increase the chances of permanent long-term damage [74], including cognitive decline and dementia. While further research is necessary to clarify cause and effect relationships, particularly the extent to which a genetic predisposition is necessary, breaking the inflammatory cycle and/or hippocampal neurogenesis is likely to be beneficial for preventing both depression and dementia.

Both acute and chronic responses to exercise have been identified, including increases in atrial natriuretic peptide (ANP), brain natriuretic peptide (BNP), copeptin, and growth hormone, as well as chronic adaptations of copeptin and thiobarbituric acid reactive species (TBARS) [89]. In a recent review of the neurobiological effects of exercise on depression, Schuch et al. [89] reported that the mechanisms by which exercise affects depression are yet to be clearly understood. Despite limited evidence that exercise promotes neurogenesis and changes in inflammation biomarkers and overall brain structure, two studies included within the review reported evidence of an association between improvements in depressive symptoms and increases in hippocampus volume and IL-1B following exercise [89].

At the same time, a recent systematic review and meta-analysis confirmed that yoga has a downregulating effect on the sympathetic nervous system and the HPA axis in response to stress, including reductions in morning cortisol, blood pressure, and heart rate [48]. There is also preliminary evidence suggesting yoga may increase BDNF [90]. Mindfulness-based psychological therapies which have some overlap with yoga (e.g., mindfulness-based stress reduction (MBSR), mindfulness-based cognitive therapy (mbCT/mCBT/MBCT)) are known to downregulate the sympathetic nervous system and the HPA axis in response to stress [64, 91–94] and are associated with long-term increases in hippocampal grey matter [95]. Beyond these anti-inflammatory effects, yoga and related activities such as mindfulness meditation may improve mood and cognition via increased focus, attentional awareness, and emotion regulation and/or reduced neuroticism or negative perseverative thinking (i.e., rumination, worry) [96–100]. In this way, yoga combines both top-down (from mind to body, through mindfulness) and bottom-up (from body to mind, through reduced neuroinflammation and stress response) effects that may distinguish it from exercise, where top-down effects are a potential side-effect versus core component. Further research is needed to disentangle the unique versus shared mechanisms of action between exercise and yoga.

## 7. Implications for Treatment

Henceforth, the challenge ahead lies in the adaptation and translation of findings from clinical trials to person-centred initiatives, capable of delivering appropriate exercise and yoga interventions at a scalable level for people at risk of or experiencing depression and/or cognitive decline. Despite the numerous protective and treatment benefits that physical activity and yoga may offer, effectiveness of these interventions is overwhelmingly limited by the barriers to being physically active that are prevalent across society (i.e., poor motivation) and compounded among those experiencing symptoms of depression or cognitive decline [101]. While continued research into the mechanisms behind the antidepressive effects of exercise or yoga is certainly required, a focus on implementation and addressing cultural, educational, and logistical issues within treatment services is equally as important. While behavioral activation, a key component of CBT that involves scheduling activities that are pleasurable and allow opportunities for mastery [102–104], may include some form of physical activity, this review suggests that it is necessary. From a pragmatic perspective, clinical psychologists may combine this scheduling with positive data logs [105] and gratitude or savoring techniques [106–110] to increase compliance. Furthermore, programs delivered by professionals such as physiotherapists and exercise physiologists with tertiary training in exercise prescription are associated with reduced dropout and increased motivation to exercise among people with an affective disorder [111], highlighting the importance of multidisciplinary mental health teams to ensure the translation of findings into real world, scalable programs [112–114].

## 8. Future Directions

Important questions remain. In particular, it is currently unclear whether or not there are unique or combined effects of physical activity versus mindfulness/meditation. That is, is there something extra special about yoga (which combines the two, within the same activity), or can either individually exert similar positive benefits? Alternatively, is there an external third factor (e.g., regular routine, organised social activity) contributing to the improvements? Currently, there is a paucity among the literature. Very few studies have compared yoga to either exercise or mindfulness/meditation as short-term interventions, for either mood or cognitive deficits. Beyond that, no studies have compared the long-term effects of any of these interventions in the prevention of either mood disorders or clinical cognitive deficits.

The heterogeneity of depression also needs to be addressed. Indeed, individuals require 5 of a possible 9 diagnostic symptoms to meet criteria for MDD, creating high internal variability amongst individuals, independent of symptom severity (see [115] for further discussion). Furthermore, do factors such as clinical symptom severity (including comorbidity) or clinical course (recurrent versus chronic, age of onset, etc.) moderate the potential benefits of exercise and/or yoga? For example, depression and anxiety are highly comorbid [116–120], which negatively affects treatment and recovery, quality of life, and global functioning, over and above the effects of either disorder independently [121–123], suggesting comorbid depression/anxiety may be quantitatively or qualitatively different than either disorder alone. Studies have also demonstrated that anxiety negatively affects cognition [2, 3, 124, 125] and that exercise [26, 46] and yoga [46, 126] may help to ameliorate these associated cognitive deficits. However, no study to date has teased apart the unique effects of exercise and/or yoga on anxiety versus depression, in relation to risk for cognitive decline. Furthermore, while it is known that age of onset of depression plays a role in the development of dementia [8, 10–13], it is unknown how this interacts with treatments. For example, is there crucial or optimal time window for intervention? Future studies should investigate the potential benefits of exercise and yoga in the context of clinical severity and course.

## 9. Conclusion

Depression is a potentially modifiable risk factor for dementia. Both exercise and yoga are effective treatments for depression and cognitive decline that are also relatively easy and cost-effective to implement. However, it is currently unclear which components of these interventions (e.g., physical activity, mindfulness) are necessary or sufficient to produce change. Further research is also needed to determine whether these interventions are capable of preventing or at least slowing the development of clinically significant deficits in cognitive function and, if so, the optimal timing of these interventions.
